# A Comparison of the Effects of Supervised versus Home Schroth Exercise Programs with Adolescent Idiopathic Scoliosis

**DOI:** 10.3390/children11030354

**Published:** 2024-03-17

**Authors:** Kadriye Tombak, İnci Yüksel, Umut Ozsoy, Yılmaz Yıldırım, Sezen Karaşin

**Affiliations:** 1 Department of Physical Therapy, Vocational School of Health Services, Akdeniz University, Antalya 07058, Turkey; 2 Department of Physiotherapy and Rehabilitation, Faculty of Health Sciences, Eastern Mediterranean University, Famagusta 99628, Turkey; iyuksel@hacettepe.edu.tr; 3 Department of Anatomy, Faculty of Medicine, Akdeniz University, Antalya 07058, Turkey; ozsoyu@akdeniz.edu.tr (U.O.); yildirimyilmaz@akdeniz.edu.tr (Y.Y.); ssezen.k@gmail.com (S.K.)

**Keywords:** scoliosis, posture, quality of life, trunk asymmetry, 3D surface topography

## Abstract

(1) Background: Schroth exercise can reduce the deformity of the spine and improve the life quality and the body image of patients with adolescent idiopathic scoliosis (AIS). (2) Methods: The study began with 49 participants, aged 10–16 years old, who were diagnosed with AIS. At the end of the study, 37 patients were randomly assigned to either the Supervised (n = 19) or Home-Based Schroth Exercise Group (n = 18) and completed the study. Both groups were treated for seven days a week over twelve weeks. For all patients, body rotation measurements were performed with a scoliometer, surface asymmetry analysis was carried out using an Artec Eva 3D scanner, health-related quality of life was evaluated by the Scoliosis Research Society-22 (SRS-22) questionnaire, and the perception of the cosmetic deformity was assessed by the Walter Reed Visual Assessment Scale (WRVAS). All the measurements were repeated before and after the 12-week treatment. (3) Results: Post-treatment scoliometric measurements showed a significant decrease in body rotation in both groups (*p* < 0.05). Similarly, both groups observed significant positive changes in SRS-22 and WRVAS scores (*p* < 005). RMS values were statistically significant in both groups; the difference was only statistically significant in the thoracic anterior arm subparameter (*p* < 0.05). (4) Conclusion: The Schroth exercise for both groups with AIS improved body symmetry, quality of life, and body image.

## 1. Introduction

Scoliosis is a three-dimensional change in the spine’s alignment, including structural spine changes affecting the sagittal, coronal, and horizontal planes [1,2]. Adolescent idiopathic scoliosis (AIS) occurs in the pubertal growth phase (from 10 to 16 years of age) and sometimes progresses rapidly; studies have shown that in adolescents, the prevalence of AIS is 5.2%, and the annual incidence is 2% [1,3,4]. AIS causes asymmetries in the musculoskeletal system and, consequently, morphological and geometric changes in the trunk. Numerous problems, such as postural changes, sensory disturbances, balance and gait problems, physical activity limitations, pain, body image disorders, and social communication disorders, occur in individuals with AIS [3,5,6,7,8].

Since the progression of AIS can be slowed or even stopped with early diagnosis and proper management, conservative treatment (exercise and bracing) becomes essential, especially in curvatures up to 40 degrees [9,10,11]. Furthermore, there is increasing evidence of the effectiveness of exercises [11,12,13,14]. Therefore, the term Physiotherapy Scoliosis Specific Exercises (PSSEs) is used for exercise concepts explicitly developed for scoliosis in the published literature [15,16,17,18,19]. Evidence is also growing regarding exercises, with former Cochrane Review results reporting that the currently very low-quality evidence for this treatment is expected to change. A recent systematic review reported that PSSEs can reduce spinal deformities and improve quality of life as an isolated treatment or when administered in addition to bracing [14,20,21].

The Schroth exercise method aims to achieve symmetrical loading with isometric muscle contractions and different exercises to strengthen and lengthen asymmetrically developing muscles. In Schroth treatment programs, the aim is to reduce the curvature in the spine by using postural corrections with the help of internal (3D rotational breathing, joint position sensing) and external stimuli (rice bags, positioning, mirror assistance, tactile and auditory stimuli by a therapist) [10,22]. Treatments are generally carried out in individual sessions in clinics under the guidance of a physiotherapist. Also, group sessions are organized in boarding camps, or exercises are prescribed as home programs [18,22,23,24]. Individual sessions are thought to increase motivation, self-confidence, and body awareness for individuals with scoliosis. The treatment is designed so participants can adapt the techniques learned in these sessions to their daily lives. The Schroth method is often applied in clinics under physiotherapists’ supervision [12,23,25].

On the other hand, home exercise programs can be recommended as an alternative to supervised exercise programs due to their advantages, such as being inexpensive and more comfortable to access. In a study by Kuru et al., the supervised Schroth exercise program was superior to the home exercise program; moreover, it was reported that scoliosis progressed in participants following home exercises [10,26]. The effectiveness of home exercises is unknown because it is difficult for the family to control home exercises or adolescents do not take the situation seriously. However, for individuals who do not have access to individually planned Schroth exercises, the home program can sometimes be a “necessity”. There are not enough studies in the literature evaluating the effectiveness of home exercises with well-educated families, individuals with scoliosis, and objective data. In a study by Negrini and colleagues, even though the Scientific Exercises Approach to Scoliosis (SEAS) exercises may seem simple because they require less physiotherapist supervision and use fewer home exercises prescribed at lower doses than some of the other scoliosis-specific exercise approaches, their effectiveness was reported when combined with real expertise in exercises, patient, and family management [19].

The present study aimed to compare the effects of three-dimensional (3D) Schroth exercises given under the supervision of a physiotherapist or as a home program for 12 weeks with surface topography and questionnaires.

The hypothesis of the study was that in adolescent idiopathic scoliosis, controlled Schroth exercises would better improve the effects of trunk symmetry, trunk topography, health-related quality of life, and perception of cosmetic deformity compared to home-program methods.

## 2. Materials and Methods

### 2.1. Participants

Referrals were obtained from the university hospital’s physical medicine and rehabilitation clinic, where an idiopathic scoliosis diagnosis was confirmed by taking x-ray images. The inclusion criteria included diagnosis by a doctor as having adolescent idiopathic scoliosis according to the Risser classification, being between the Risser scores of 0 and 3, incomplete spinal skeletal maturity, and agreeing to a 12-week treatment period. Exclusion criteria for participation in the study were a tumor; rheumatological, cardiovascular, or pulmonary disease; a neurological impairment causing mental retardation; being on an additional conservative treatment for scoliosis; having undergone spinal surgery for any reason; or refusal to sign the relevant consent form. The participants’ parents signed an assent form to agree to their child’s participation in the study.

### 2.2. Study Design, Sample Size, and Randomization

This study was planned as an evaluator-blind, randomized, controlled study. In addition, a study by Kuru et al. (2015) was taken as a reference [10]. To determine the study’s sample, version 3.1.9.4 of the G*Power program (Heinrich-Heine-Universität Düsseldorf, Germany) was used [27]. A probability level of *p* < 0.05 was chosen as the statistically significance level. Means were given with a 95% confidence interval (95% CI). Moreover, determining the sample size showed a need for 32 participants, with at least 16 in each group. To obtain 80% statistical power (1 − β error probability) with an α error level probability of 0.05, we performed repeated-measures analysis of variance (ANOVA), with in-between interaction and a medium effect size of 0.26. Then, the participants were randomly allocated to their treatment using random numbers. Numbered envelopes, prepared by the researcher who was blinded to the study and responsible for the data analysis, provided randomization with a computer-generated sequence using the “Research Randomizer” website software (https://www.randomizer.org/, accessed on 20 April 2018) [28]. Since there was no pre-defined participant pool, participants chose envelopes in order of arrival. A total of 49 adolescents with idiopathic scoliosis were randomly divided into the Supervised Schroth Exercise Group (SSEG, n = 25) and the Home-Based Schroth Exercise Group (HSEG, n = 24). Considering a dropout rate of 15% and aiming to increase the statistical power of the results, 37 participants were recruited into the study.

### 2.3. Outcome Measures

The Schroth physiotherapist recorded the demographic data of the participants. The second physiotherapist, who was unaware of the group allocation (blinded), carried out all other measurements. All the evaluations performed before starting the treatment were repeated at the end of the 12-week treatment program.

The body rotation angle (ATR) was measured by a scoliometer. In addition, a specially designed inclinometer was used to measure one part of the clinical deformity (trunk asymmetry) [29,30]. Measurements were made without shoes and with the feet parallel to each other in a forward-bent position. During the measurement, the participants were asked to bring their hands together in front of their trunk to keep the scapula and pelvis position stable and bend forward without bending their knees. Each measurement was repeated three times, and the highest value was recorded [29,30,31].

**Figure 1:**
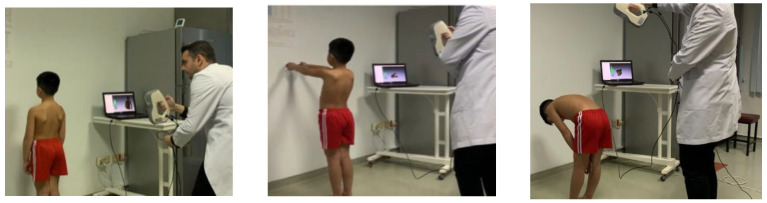
Scanning process in three different positions with the Artec EVA 3D scanner (Artec Group 2013, Luxembourg).

The geometry of the back’s surface was digitized with the Artec EVA 3D scanner (Artec Group 2013, Luxembourg) [26,32]. Each participant was asked to remain as still as possible during the scan for the best quality screening. Therefore, during the scan, the participant’s back was left completely exposed, and long hair was kept out of the way to avoid spoiling the scan. Topographic measurements were performed from the back, breathing normally, with the participant in three different body positions to record the whole back (Figure 1): (P1) in a natural upright standing position, with the arms at the side of the body; (P2) standing upright, with the arms extended straight forward; and (P3) during forward bending (as per the Adams forward bend test). The first and second positions represent the body positions used in radiographic imaging, while the third position is the body position used for vertebral rotation measurement [33,34].

The working scanning distance recommended by the scanner’s manufacturer was from 0.4 to 1 m, and the three-dimensional accuracy was reported as up to 0.05 mm at a resolution of 0.1 mm [26]. Scanning took approximately 20 s for each.

**Figure 2:**
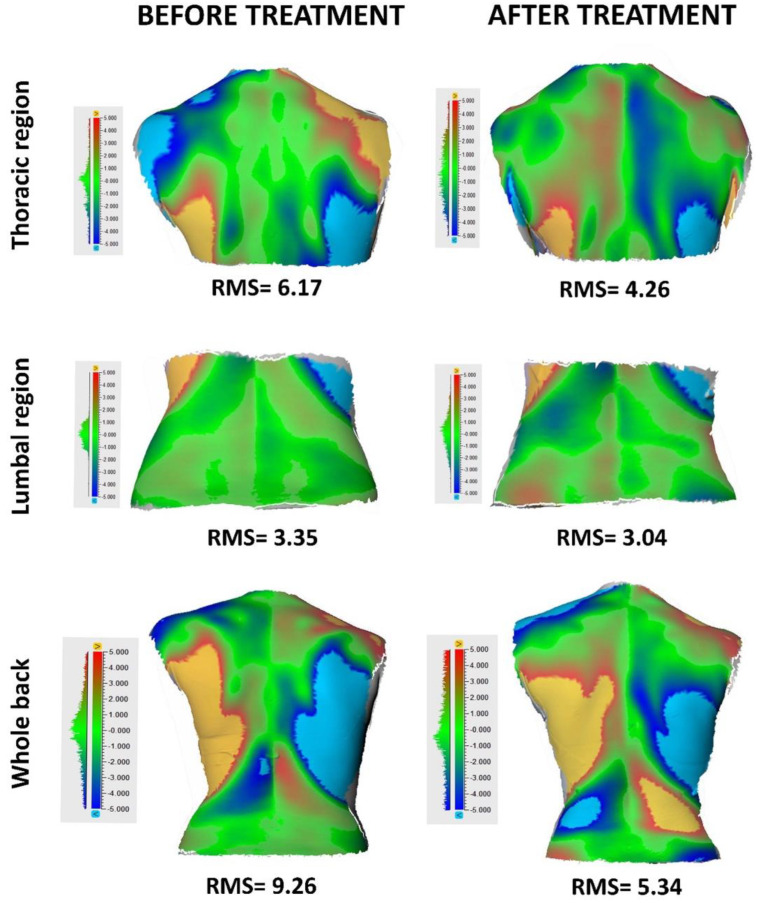
Calculation of the root mean square (RMS) values: the images were aligned and overlapped using the Artec Eva studio software (version 9.2.3.15) before and after treatment.

Three-dimensional images of the back (Figure 2) were obtained to evaluate surface asymmetry. Before scanning, the patient’s spinal processes were marked with a skin pen. After scanning, the digitized image of the back surface was divided from curvature levels into thoracic and lumbar segments. The projections of each vertebra’s spinal processes were marked on the skin before scanning, and they were used to divide the back region into thoracic and lumbar segments. The thoracic region was determined as the surface between the horizontal planes passing through the spinose processes of the first cervical and twelfth thoracic vertebrae. The lumbar region was determined as the surface between the horizontal planes passing through the spinose processes of the twelfth thoracic and first sacral vertebrae. Afterward, the 3D mirror images of the thoracic or lumbar region were created from these images using Netfabb Basic 6.0.0146 (n-Netfabb GmbH 2013 Lupburg, Germany) software. The root mean square (RMS), which was the asymmetry value, was calculated by aligning the original and mirror images of both sides of thoracic or lumbar sections with the Artec Eva studio program and overlapping them (Figure 2). Both sides of the region of interest were included in the calculation. The RMS values obtained quantitatively showed the asymmetry between the right and left halves of the back. Each back surface has an average of thirty thousand points (vertices). Therefore, the geometrical differences between the two surfaces can be calculated by the RMS of the distances between the points forming these surfaces [35,36,37].

In a previous study, the reliability of the surface topography method was shown for both intraobserver (r, P1: 0.96, P2: 0.99, P3: 0.93) and interobserver correlations (r, P: 0.84, P2: 0.97, P3: 0.96). The standard errors (SEs) of observer 1, first scan (SE, P: 0.13, P2: 0.06, P3: 0.2); observer 1, second scan (SE, P1: 0.11, P2: 0.28, P3: 0.16); and observer 2 (SE, P1: 0.09, P2: 0.28, P3: 0.19) were also determined. In addition, the correlation between this methodology and classical methods used in scoliosis was investigated. The RMS and Cobb values in all three body positions in the thoracic (r, P1 = 0.80, P2 = 0.76, P3 = 0.71) and lumbar regions (r, P1 = 0.56, P2 = 0.65, P3 = 0.63) were found to be significantly (*p* < 0.0001) correlated. Similarly, a significant (*p*  < 0.0001) correlation was found between RMS and scoliometer values in the thoracic (r, P1 = 0.58, P2 = 0.50, P3 = 0.41) and lumbar regions (r, P1 = 0.35, P2 = 0.41, P3 = 0.59) [35].

Quality of life was evaluated using the adapted Turkish version of the Scoliosis Research Society-22 (SRS-22) questionnaire, for which a Turkish validity and reliability study has been conducted [38]. The SRS-22 evaluates health-related quality of life in five domains using 22 questions. These five domains are pain, image/appearance, function/activity, mental health, and treatment satisfaction. A score between 0 (worst) and 5 (best) was given for each question. We calculated the total score. A high score indicated an increase in quality of life [38]. The Walter Reed Visual Assessment Scale (WRVAS), developed for participants with idiopathic scoliosis, was used to evaluate the perception of cosmetic deformity. WRVAS is a valid method to evaluate the perception of cosmetic deformity [39]. The WRVAS consists of seven figures evaluating the severity of asymmetries, such as spinal curvature, rib prominence, flank prominence, deformity/alignment of the thorax to the pelvis, trunk imbalance, shoulder asymmetry, and scapular asymmetry. Each of the seven figures consists of five different pictures. The participant scores these images between 1 (minimum asymmetry) and 5 (maximum asymmetry). The score evaluates perceived appearance, such as how the participant feels and how others perceive them [39,40]. The total score ranges from 7 to 35. A high score indicates severe deformity.

### 2.4. Interventions

The treatment programs for both groups were carried out by a physiotherapist (first author) who completed the International Schroth Three-Dimensional Scoliosis Therapy training and certification. Another physiotherapist (third author), who did not know which group the participants belonged to, conducted the evaluations.

#### 2.4.1. Schroth Exercises Procedure

**Figure 3:**
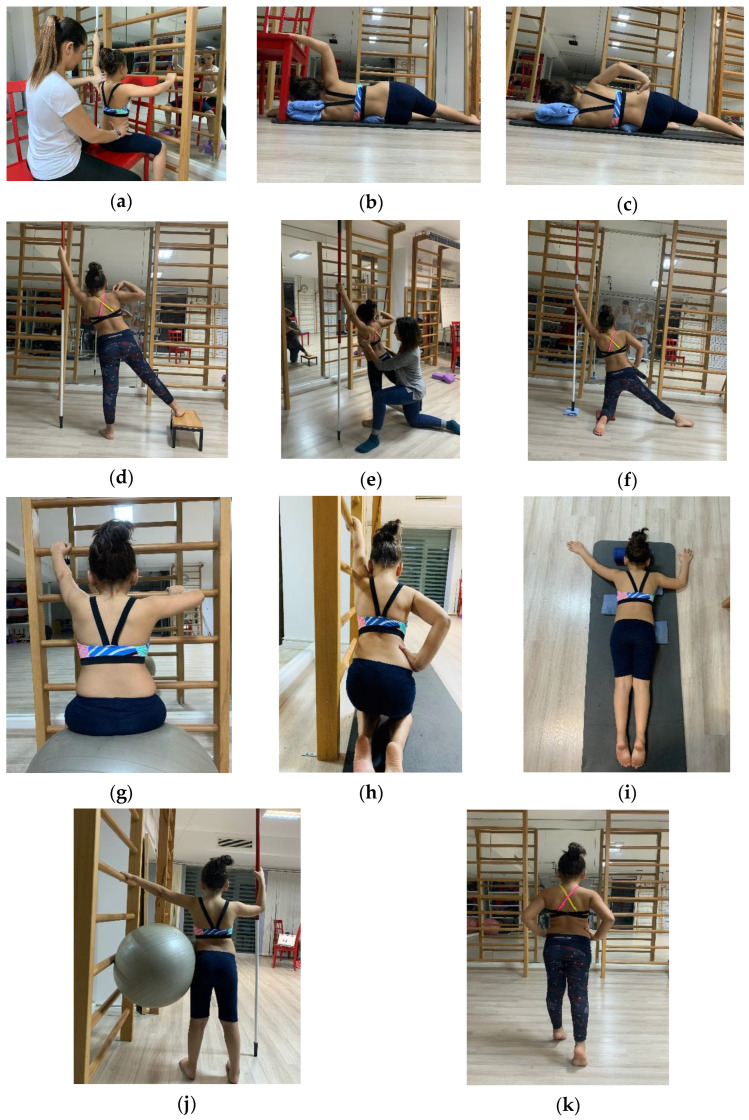
Schroth exercises: (**a**) rotational respiration and elongation study, (**b**) shoulder counter-traction lying on the side, (**c**) muscle cylinder exercises lying on the side, (**d**,**e**) sail exercise, (**f**) muscle cylinder exercises in the half-kneeling position, (**g**) sitting on a ball, (**h**) sideways hangs, (**i**) shoulder counter-traction in the prone position, (**j**) prominent hip exercise, (**k**) Schroth gait exercises.

In the first training session, the participants from both groups were trained on Schroth’s basic principles, including 3D Schroth rotational breathing exercises (Figure 3), pelvic correction exercises, elongation, fundamental tension, and positioning. During the same session, participants were taught shoulder counter-traction, muscle cylinder, sideways hangs, sail, prominent hip, and Schroth walking exercises [18,23]. The classification was made according to four different curve types developed by the Schroth method [41]. The thoracic overcorrection position was only used in groups with major thoracic curvature.

#### 2.4.2. Supervised Schroth Exercise Group (SSEG)

Participants involved in the SSEG (n = 25) undertook their individual 3D Schroth exercise program under a physiotherapist’s supervision for one hour twice a week for twelve weeks. Participants in this group exercised at home for the remaining one hour five days a week. The first session was an hour, and the participants and their families were informed about the treatment program. The exercise program was created from 3D Schroth and breathing exercises that fit the individuals’ scoliosis curvature patterns. The number of 3D Schroth exercises initially given (8 to 10) was increased to 15 different exercises when doing the exercise in the 12-week treatment period. For each exercise, there were five repeats and there were two sets for sessions. The exercise process progressed from basic to complex exercises. On other days, the individuals did the same exercises at home with video recordings that were prepared for them and filled out the exercise follow-up forms. Family members were also aware of the participants’ need to continue the exercise and checked the exercise follow-up forms. The same physiotherapist treated all subjects with AIS individually in the study.

#### 2.4.3. Home-Based Schroth Exercise Group (HSEG)

The individuals in the HSEG (n = 24) were asked to do a home program consisting of 3D Schroth exercises for about 1 h every day for 12 weeks. The physiotherapist designed a home training program in the initial session and practiced with the patients for approximately 90 min. This training taught 3D Schroth and breathing exercises according to the individuals’ scoliosis curve shapes, like in the SSEG. The number of 3D Schroth exercises initially given (8 to 10) was increased to 15 different exercises during the 12-week treatment period. For each exercise, there were five repeats and there were two sets for sessions. The exercise process progressed from basic to complex exercises. The Schroth therapist recorded the exercise session on the patient’s phone or tablet to help them remember the home program correctly.

The physiotherapist checked the participants’ exercises every four weeks in the clinic, and necessary corrections and additions were made. The same physiotherapist carried out exercise training and weekly controls for all individuals. To increase cohesion, home equipment and access to facilities were provided, it was made fun by technology support/video recording, and parent involvement was encouraged. When the compliance fell below 83%, attempt was made to solve the problems in cooperation with the patients and families. The movements given in the corrected positions were repeated until the participants made the movements correctly. When the participants accurately completed one set and five repetitions, corrections in other positions continued. Participants recorded the date and time (minutes) of the exercise sessions on the follow-up forms. Our most important criterion for making exercise changes was that the participant was able to do the exercise correctly and adequately. It was also essential to adapt the correction to their daily life. Regularly, our main criteria were beyond the number of repetitions or the duration.

### 2.5. Statistical Analyses

The statistical analyses in the study were performed using SPSS software version 24. The variables’ suitability to normal distribution was examined using visual (histogram and probability graphics) and analytical methods (Shapiro–Wilk tests). Descriptive analyses were presented using mean and standard deviations for normally distributed variables. Two-way analysis of variance (mixed-design repeated measures ANOVA) was adopted in repeated measures to evaluate the changes in time and the group–time interactions of the variables of the SSEG and HSEG groups, as determined by the measurements. The total type-1 error level was determined as 5% for statistical significance.

## 3. Results

**Figure 4:**
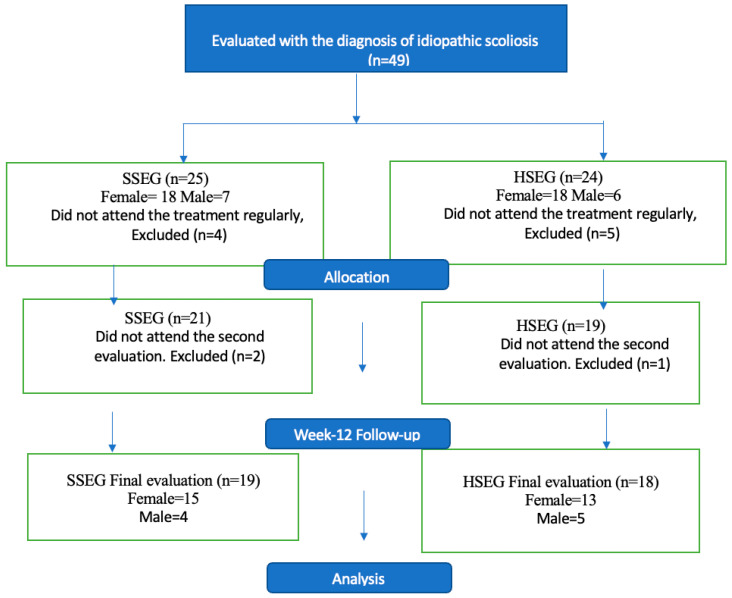
Participant enrollment and study flowchart. Abbreviations: SSEG: Supervised Schroth Exercise Group, HSEG: Home-Based Schroth Exercise Group.

A total of 37 participants (SSEG: n = 19 and HSEG: n = 18) completed all the evaluations and the 12-week treatment protocols (Figure 4). The study included 28 female and 9 male participants. Table 1 gives the general characteristics of the participants.

Exercise compliance was calculated separately for both groups. The compliance value for accepted participation was calculated as 83.3%. For SSEG, exercise compliance performed with a physiotherapist was 100%, and home exercise compliance was 97.54%. In the home group (HSEG), compliance with the home program was 96.89%. The compliance of participants who dropped out of the study was zero. The participants’ attendance is detailed in a flow chart (Figure 4). Forty-nine participants started, but twelve (SSEG n = 6, HSEG n = 6) were excluded from the study for some reason (did not participate in the last measurement or attend the treatment regularly).

According to the Schroth classification, the pelvic shift was in the direction of the thoracic concavity in 16 participants and the lumbar concavity direction in 3 participants within the SSEG (n = 19). On the other hand, in the HSEG (n = 18) participants, the pelvic shift was in the direction of the thoracic concave curve in 14 participants and the direction of the lumbar concavity in 4 participants. This was an essential point for us to create personalized exercise programs.

### Comparison between Differences and Changes in SSEG and HSEG during Treatment

Before treatment, a significant difference (*p* < 0.05) was observed between the surface asymmetry (RMS) values in the thoracic region in all bodies. There was no significant change in the surface asymmetry (RMS) values in the lumbar regions and the whole back. After treatment, a significant difference (*p* < 0.05) was found in the thoracic region in the standing and arms-forward positions. For the RMS estimates for the thoracic region in the standing position, both groups experienced significant improvements over time (main effects of time: *p* = 0.022). However, the interaction between the group and time did not reach significance, suggesting that the effect was not significantly more significant in one group than the other. The main effects of time on the thoracic RMS in the bending forward positions were *p* = 0.010, but the interaction between group and time did not reach significance, suggesting that the effect was not significantly more significant in one group. The main effects of time on the thoracic RMS in the arms-forward position were *p* = 0.009, and the interaction between group and time reached significance, suggesting that the effect was significantly more significant in one group than the other (*p* = 0.017). Only the thoracic RMS improved more in the SSEG than in the HSEG over time.

Otherwise, both groups improved over time in the other two thoracic RMS variables: the lumbar RMS with bending forward and the thoracolumbar RMS in standing and bent-forward positions (Table 2).

In the comparison of scoliometer (thoracic and lumbar) and SRS-22 scores, no significant difference was found between pre-treatment (*p* > 0.05) and post-treatment (*p* > 0.05) values. On the other hand, in the comparison of WRVAS scores, no significant difference was found before treatment (*p* > 0.05), but there was a difference after treatment (*p* < 0.05). A significant decrease was observed in scoliometer measurements in the thoracic and lumbar regions in the SSEG (*p* < 0.001) and the HSEG (*p* = 0.001). A significant difference in SRS-22 and WRVAS scores was observed in the SSEG (*p* < 0.005) and HSEG (*p* < 0.005) (Table 3). For the scoliometer, SRS-22 and WRVAS measurements for both groups experienced significant improvements over time (main effects of time: *p* < 0.01), but the interaction between group and time did not reach significance, suggesting that the effect was not significantly more significant in one group than the other (*p* > 0.05) (Table 3).

## 4. Discussion

In this study, significant improvements were observed in both groups in terms of body rotation, SRS-22 scores for quality of life, and WRVAS scores for deformity perception after treatment. Surface topography measurements indicated improvements in the thoracic region for all three positions and significant changes in lumbar and whole-back positions. The present research confirms the effectiveness of Schroth and Schroth-based exercise programs in treating scoliosis, aligning with previous studies reporting reduced curvature progression, lower surgical rates, improved quality of life, enhanced body image, increased muscle strength and tone, and improved vital capacity [3,8,14,15,25,42,43,44,45]. The 3D exercise results of this study supported the existing literature, and although Cobb angle results were unavailable due to short exposure intervals, improved quality of life and body image perception were observed. Randomized controlled studies have reported that Schroth exercises reduce the Cobb angle to level II and improve quality of life [14,46]. Additional studies cited herein support the positive impact of scoliosis-specific exercises, especially when supervised by a physiotherapist [47]. The present study’s findings highlight the potential for using surface topography in situations where repeated X-rays are impractical, achieving improvements in trunk rotation, quality of life, and body image perception within a shorter exercise period. The significant changes observed in the SRS-22 and WRVAS scores for both groups in a short time suggest the potential for more substantial differences with extended exercise periods.

Our study is the first in the literature on this aspect. Although the gold standard in publications is the Cobb angle, it may be necessary to report the treatment’s effectiveness with more frequent and objective data by physiotherapists. Studies on different techniques developed to determine the treatment’s efficacy are still ongoing [48,49,50,51]. Although radiographic methods such as the EOSTM system have been developed to reduce adolescent groups’ ion exposure, the importance of topographic measurements—one of the methods developed for post-diagnosis follow-up—is increasing daily [36,50]. In the published literature, studies investigating the effectiveness of the Schroth exercise method indicate that treatment durations are not long enough, and studies with more comprehensive evaluations are called for [10,15,43,52]. Komeili et al. (2015) examined the relationship between three-dimensional trunk asymmetry analyses using torso scan images and spine evaluations via radiographic images in 100 scoliosis patients. They assessed curvature number, direction, and location, as well as the location and severity of the apical vertebra. The study compared the accuracy of these methods, finding accurate determinations of curvature direction on color maps [26]. The accuracy rates for estimating curve numbers were 62%, 66%, and 23% for single, double, and triple curves, respectively, while the accuracies of location estimation were 63%, 92%, and 62%. The authors concluded that three-dimensional scanning is a reliable method for determining curve parameters. In contrast, our study focused on assessing the effectiveness of exercise using surface topography. This study, to our knowledge, is the first to use 3D morphometric asymmetry analysis to track the effects of exercise. Participants primarily had major curvatures in the thoracic region, and after physiotherapy, thoracic region RMS values significantly decreased compared to pre-treatment values (Table 2).

Surface topography analysis is a method that quantitatively reflects the change in morphology observed by the participants and their families, unlike Cobb angle measurement, which analyses the curvature of the spine and can only be evaluated by a physician. Therefore, the data obtained using this method may be very closely related to the participants’ health-related quality of life [53,54]. The present study investigated the Schroth exercise method’s effectiveness on health-related quality of life and deformity perception through questionnaires. Schroth exercises have also been reported to improve health-related quality of life parameters such as functionality, satisfaction, and pain [3,8,43,55]. According to our results, the quality of life increased significantly in both groups when Schroth exercises were given. The results of our study also support the effects of Schroth exercises on topographic change and change in the quality of life. Although stopping the progression of curvature is very valuable in rehabilitation processes, we can say that topographic and quality-of-life changes are also very effective in terms of motivation and continuity. It should not be forgotten that improvement occurs as the adolescent grows and progression continues. In addition, Cobb angle measurement, which is the gold standard used by healthcare professionals, can be performed at certain time intervals according to the adolescent’s progress and needs. Especially, annual and semi-annual measurements can reduce motivation. With these interim evaluations performed every 2 months and every 6 months for these individuals, the health professional, the adolescent, and the family can be more motivated.

The cosmetic asymmetry caused by scoliosis, which is a three-dimensional effect on the torso’s appearance, is a significant problem that concerns participants, their families, and physiotherapists [7,8,20]. A strong correlation between the perception of appearance and the curve’s radiological magnitude was reported. Pineda et al. showed that WRVAS scores correlated significantly with Cobb values (correlation coefficients, 0.4 to 0.7) [39]. According to Sanders et al., WRVAS scores strongly correlate with curve magnitude [39,40]. Studies investigating sensorimotor involvement and body awareness in AIS participants have stated a dysfunction in these individuals’ sensorimotor mechanism compared to healthy individuals [20,56]. The present study investigated the effects of Schroth exercises on the body image of participants with AIS. The results showed that body image perceptions evaluated by the WRVAS after the Schroth training program were significantly improved in both groups.

Both SRS-22 and WRVAS measurements are subjective methods accepted in the literature. In our study, we supported the efficacy of subjective measurements with quantitative 3D morphometric data. Our study will contribute to the literature on this aspect. Due to the study’s limited duration, long-term effects of the application of exercise on adolescent idiopathic scoliosis could not be investigated. However, we plan to investigate the effectiveness of exercise for more extended periods in future studies.

Working with two randomized groups is one of the strengths of our study. Differences in pre- and post-test results in intra-group interactions are shown in Table 2 and Table 3. Considering the superiority of the difference between the groups, we only observed a significant value in the thoracic RMS values in the arms-forward position. Although there was no statistically significant difference in the remaining body positions and regions in the SSEG, they can be considered clinically significant because of the larger observed changes.

In our study, because special attention was paid to the participants being highly motivated by the exercise program from the beginning of the training session, it took a certain amount of time to teach the Schroth method. We know from the literature and our experience that teaching Schroth’s principles in one session can sometimes be challenging. We have experienced this, especially with children treated with a home exercise program.

The present research revealed that trunk symmetry values improved significantly compared to pre-treatment levels. In our investigation, even though the post-treatment values of both groups were much higher than their pre-treatment values, it was determined that the treatment methods applied to the two groups were not superior to each other. This research demonstrates that Schroth therapy, whether administered under the supervision of a physiotherapist or as accompanied by a well-programmed controlled home-based Schroth exercise program, has similar positive effects. This is the only such research using comparable evaluation methods and evaluating trunk asymmetry that was discovered in the literature. Therefore, the results obtained could not be compared with other studies. In our study, in which we started to determine which method is more effective, we found this result: it was found and supports the literature that the Schroth exercise method new studies are needed, is effective if applied correctly, controlled, and used appropriately.

There are also limitations. Factors such as body fat ratio, posture, and thoracic deformity also affect the formation of asymmetries due to scoliosis [57,58]. Therefore, the most significant limitation of our method is that it analyzes not only the change caused by the curvature of the spine but also the surface deformity caused by the effects of the other mentioned factors. This influence was minimized in our study, and we studied patients with similar body mass indexes and within the normal range. The most important limitation of the surface topographic measurement method used in this study is the relatively long imaging time. During 3D scanning, each view takes approximately 20 s. The participant must maintain his or her position during the measurement. The SRS-22r is the most frequently used questionnaire assessing the quality of life in patients with AIS after treatment. Due to high ceiling effects and scores close to the best values, we suggest that different quality-of-life tools should be used for patients with conservatively treated AIS. Also, receiving treatment with the same therapist could positively influence the self-reported outcomes of patients. Also, we planned monthly controls for the HSEG in our study. However, a visit every month is still a notable amount of supervision. The topography methods utilized in our study provided us with objective data. The data obtained from the participants in our surveys were subjective, as indicated by the RMS values.

Furthermore, our study was limited in that we could not examine the Cobb angle due to ethical and clinical reasons that prevented us from repeating the radiography. Although the lack of a healthy control group and lack of longer-term follow-up are other limitations of our study, we aim to increase these evaluations and interventions in our future studies with more participants. In our study, in which we started to determine which treatment method was more effective, the need for a healthy control group appeared as a limitation.

In our investigation, both Schroth therapy procedures applied to AIS patients had successful outcomes for most of the examined parameters. However, there are few physiotherapists with Schroth training. Patients with AIS have restricted access to this therapy due to several factors. The present epidemic has hampered several health systems and made it challenging to perform treatments requiring continuity, such as physical therapy, in clinics.

Considering the results of our study, we can conclude that the supervised Schroth exercise program has a more remarkable impact on improving morphometric and cosmetic effects of AIS than the home exercise program. The home exercise program can be an alternative treatment if corrected by a physiotherapist at specific intervals.

## 5. Conclusions

In recent decades, knowledge of idiopathic scoliosis and its treatment has grown, thanks to broader interest in and better quality of research. If three-dimensional scoliosis-specific physiotherapists, such as Schroth, are unavailable, alternative methods should be investigated today. We recommend that future clinical studies examine the effects of different types of exercise techniques. The literature needs more clinical studies.

## Figures and Tables

**Table 1:** Comparison of the baseline characteristics.

	Group		*p*
	SSEG (n = 19) X ± SD	HSEG (n = 18) X ± SD	
Age (years)	13.47 ± 1.71	14.16 ± 2.03	0.89
BMI (kg/m^2^)	21.12 ± 1.51	21.22 ± 1.50	0.845
Cobb Thoracic (angle)	18.52 ± 9.94	14.14 ± 5.49	0.109
Cobb Lumbar (angle)	16.13 ± 8.54	11.83 ± 4.68	0.068
Risser	2.26 ± 1.04	2.38 ± 0.84	0.692

All measured values are presented as mean ± standard deviation, independent samples *t*-test. Abbreviations: SSEG: Supervised Schroth Exercise Group; HSEG: Home-Based Schroth Exercise Group; BMI: body mass index; X: mean; SD: standard deviation.

**Table 2:** Time and group–time comparison of RMS measurement data of SSEG and HSEG.

		SSEG (n = 19)		HSEG (n = 18)		Time	Group * Time	Effect Size
		X	SD	X	SD	F/*p*	F/*p*	
Thoracic RMS Standing (mm)	Test B	5.93	4.60	2.97	1.45	5.71/0.022 *	2.71/0.108	0.072
	Test A	4.13	1.81	2.64	1.08			
Thoracic RMS Arms Forward (mm)	Test B	5.90	4.00	2.96	1.49	7.72/0.009 **	6.27/0.017 *	0.152
	Test A	3.89	1.79	2.86	.76			
Thoracic RMS Bending Forward (mm)	Test B	4.94	3.01	3.25	2.12	7.41/0.010 *	3.17/0.083	0.083
	Test A	3.79	2.14	3.01	1.37			
Lumbar RMS Standing (mm)	Test B	3.18	1.90	2.84	2.08	2.79/0.104	0.44/0.512	0.012
	Test A	2.91	1.59	2.22	1.12			
Lumbar RMS Arms Forward (mm)	Test B	3.79	3.00	2.79	1.30	1.23/0.274	0.09/0.766	0.003
	Test A	3.25	1.98	2.48	1.20			
Lumbar RMS Bending Forward (mm)	Test B	3.72	2.17	3.05	1.70	7.99/0.008 **	1.79/0.189	0.049
	Test A	2.47	1.15	2.60	1.82			
Whole-Back RMS Standing (mm)	Test B	6.19	3.20	4.57	1.89	4.85/0.034 *	0.21/0.648	0.006
	Test A	5.39	1.88	4.05	1.42			
Whole-Back RMS Arms Forward (mm)	Test B	6.23	2.76	5.01	2.07	3.01/0.091	0.80/0.376	0.022
	Test A	5.51	2.42	4.78	2.04			
Whole-Back RMS Bending Forward	Test B	5.83	2.60	4.73	1.97	17.64/0.000 **	1.32/0.258	0.036
	Test A	4.88	2.38	4.19	1.52			

All measured values are presented as mean ± standard deviation. Two-way analysis of variance (mixed-design repeated measures ANOVA, ** *p* < 0.01, * *p* < 0.05). Abbreviations: SSEG: Supervised Schroth Exercise Group, HSEG: Home-Based Schroth Exercise Group, RMS: root mean square, B = before, A = after, X: mean, SD: standard deviation.

**Table 3:** Time and group–time comparison of scoliometer measurements, SRS-22 totals, and WRVAS data of SSEG and HSEG.

		SSEG (n = 19)		HSEG (n = 18)		Time	Group * Time	Effect Size
		X	SD	X	SD	F/*p*	F/*p*	
Thoracic Scoliometer Measurement (degrees)	Test B	6.84	3.56	6.11	2.42	84.08/0.000 **	0.59/0.445	0.017
	Test A	3.68	2.54	3.44	1.10			
Lumbar Scoliometer Measurement (degrees)	Test B	5.95	2.90	5.39	2.03	106.39/0.000 **	0.94/0.338	0.026
	Test A	3.26	2.10	3.17	1.47			
SRS-22 (points)	Test B	4.01	0.40	3.91	0.38	33.58/0.000 **	0.24/0.622	0.007
	Test A	4.22	0.31	4.16	0.33			
WRVAS (points)	Test B	17.58	4.61	19.50	4.27	75.55/0.000 **	0.17/0.680	0.005
	Test A	13.42	2.69	15.72	3.91			

All measured values are presented as mean ± standard deviation. Two-way analysis of variance (mixed-design repeated measures ANOVA, ** *p* < 0.01). Abbreviations: SSEG: Supervised Schroth Exercise Group, HSEG: Home-Based Schroth Exercise Group, SRS-22: Scoliosis Research Society-22, WRVAS: Walter Reed Visual Assessment Scale, B: before, A: after, X: mean, SD: standard deviation.

## Data Availability

The data presented in this study are available on request from the corresponding author. The data are not publicly available due to specific ethical and privacy considerations.

## Abbreviations

The following abbreviations are used in this manuscript:

AIS	Adolescent idiopathic scoliosis
SRS-22	Scoliosis Research Society-22
WRVAS	Walter Reed Visual Assessment Scale
PSSEs	Physiotherapy Scoliosis Specific Exercises
SEAS	Scientific Exercises Approach to Scoliosis
CI	Confidence interval
SSEG	Supervised Schroth Exercise Group
HSEG	Home-Based Schroth Home Exercise Group
ATR	Angle of trunk rotation
RMS	Root mean square
SD	Standard deviation
B	Before
A	After
X	Mean
